# DEVELOPING THE COMMON MARMOSET AS A TRANSLATIONAL MODEL OF AGE-RELATED OSTEOARTHRITIS

**DOI:** 10.1093/geroni/igz038.390

**Published:** 2019-11-08

**Authors:** Dennis M Minton, Angela J Marolf, Kelly S Santangelo, Adam B Salmon, Adam R Konopka

**Affiliations:** 1 University of Illinois at Urbana-Champaign, Urbana, Illinois, United States; 2 Colorado State University, Fort Collins, Colorado, United States; 3 University of Texas Health at San Antonio, San Antonio, Texas, United States

## Abstract

Age is a primary risk factor for osteoarthritis (OA). The mechanisms that contribute to OA are poorly understood and disease modifying treatments have not been identified. A critical shortcoming in developing therapies is the limited number of translational models available to identify the causes of naturally occurring OA. Our goal is to use the common marmoset as a non-human primate (NHP) model of age-related OA. NHP are the closest evolutionary relative to humans and share many characteristics of human aging. The marmoset has advantages over other NHP for aging research because of their relatively short maximal lifespan and small size. Micro-computed tomography (uCT) was performed on whole-knee joints obtained from young (10 yrs, n=3) marmosets at necropsy. OA was evaluated using a clinical uCT scoring system and quantitative assessments of subchondral bone structure and ossified meniscal volume. Advancing age was positively correlated to increased uCT OA score (p<0.05, r=0.59 ), mainly through increased number and size of osteophytes and progressive subchondral bone sclerosis from the medial to both medial and lateral compartments. For marmosets displaying meniscal ossification, older marmosets had greater (p<0.05) ossified meniscal volume than middle-aged and younger marmosets, respectively. Trabecular (p=0.05) and cortical bone thickness (p<0.05) were also lower in older marmosets. These data are the first to indicate that the marmoset develops naturally occurring, age-related OA and support the pursuit of additional studies using the marmoset to identify OA mechanisms and test potential interventions.

